# Psychological distress and self-rated health status in reproductive aged women with pain: findings from a national, cross-sectional survey

**DOI:** 10.1186/s12905-019-0757-7

**Published:** 2019-05-07

**Authors:** April M. Miller, Fiona Judd, Peter A. Dargaville, Amanda L. Neil

**Affiliations:** 1 Menzies Institute for Medical Research, University of Tasmania, Hobart, Australia; 2 Perinatal and Infant Mental Health Team, Child and Adolescent Mental Health Services (CAMHS) South, Hobart, Australia; 3 Neonatal and Paediatric Intensive Care Unit, Royal Hobart Hospital, Hobart, Australia

**Keywords:** Pain, Pregnancy, Breastfeeding, Psychological distress, Reproductive age, Women

## Abstract

**Background:**

Pain impacts upon psychological wellbeing. In pregnant and postpartum women psychological distress may negatively affect the mother-infant relationship and lead to adverse infant development. Yet, co-occurrence of pain with psychological distress in women of reproductive age has not been investigated. Therefore, this study aimed to: 1) assess prevalence of psychological distress in reproductive aged women by pain severity; and 2) examine the self-rated health status of reproductive aged women with and without pain.

**Method:**

Data for women aged 18–49 years were obtained from the 2011–12 Australian Bureau of Statistics National Health Survey. Sample data were weighted to give population estimates. Recent pain severity, self-rated health and psychological distress were analysed for pregnant, breastfeeding and non-pregnant/non-breastfeeding women.

**Results:**

Moderate-to-very severe pain was reported by 17.6% of pregnant (sample *n* = 165, weighted *N* = 191,856), 25.9% of breastfeeding (sample *n* = 210, weighted *N* = 234,601) and 23.9% of non-pregnant/non-breastfeeding women (sample *n* = 4005, weighted *N* = 4,607,140). Psychological distress was associated with pain in non-pregnant/non-breastfeeding women (*p* < 0.001). High-to-very high distress was seen in 26.4% (95% CI, 23.2–29.6) of NP/NBF, 8.1% (95% CI, 0–17.2) of breastfeeding and 7.3% (95% CI, 0–18.0) of pregnant women with moderate-to-very severe pain. Self-rated health status was associated with pain severity in pregnant (*p* = 0.001) and non-pregnant/non-breastfeeding (*p* < 0.001) women**.**

**Conclusion:**

Given the strong association between psychological distress and pain in non-pregnant/non-breastfeeding women, and the relatively common occurrence of moderate-to-very severe pain in both pregnant and breastfeeding women, assessment of psychological distress levels in all women of reproductive age who report experiencing moderate-to-very severe levels of pain may be of benefit.

## Background

Pain is an issue which affects many women internationally [1–4]. In 2012, 5–15% of women from the USA aged 18–44 reported experiencing pain ranging from ‘a lot of pain, some days’ to ‘a lot of pain, most days’, over a three-month period [3]. In Australia, approximately one in five women of reproductive age have been found to have experienced moderate-to-very high severity pain during the most recent four-week period [5]. In this same study, it was also found that 10% of non-pregnant women of reproductive age and 5% of pregnant women reported living with chronic or reoccurring pain [5].

The impact of living with pain should not be underestimated, both acute and chronic pain can negatively impact psychological wellbeing and the association is bidirectional [6, 7]. High psychological distress can be indicative of underlying depression or anxiety, both of which can have substantial impacts upon the sufferer and their family [8].

In pregnancy, pain and discomfort may be associated with normal physiological changes, and/or be due to illness or pre-existing health conditions [9, 10]. Pain experienced during pregnancy and in the postpartum period has been found to have a substantial impact upon quality of life, with small studies identifying links between postnatal depression and pain experienced during labour and in the immediate postpartum period [10–12]. Meanwhile, psychological distress can impact upon maternal-infant attachment and future childhood development [8].

Maternal psychological distress levels are modifiable, however, it is not known how pain impacts treatment success [8]. Further, pharmacological options for the management of pain in pregnant and breastfeeding women may be limited [13]. The relationship between pain and psychological distress is therefore an important consideration in women of reproductive age, but has not been thoroughly researched, particularly in reproductive aged women who are pregnant or breastfeeding. Therefore, this study aimed to: 1) assess the prevalence of psychological distress in reproductive aged women by pain severity; and 2) examine the self-rated health status of reproductive aged women with and without pain.

## Methods

The Australian National Health Survey (NHS) 2011–12 is a nationally representative survey conducted by the Australian Bureau of Statistics (ABS) across all states and territories of Australia [14]. The survey used a stratified, multistage design collecting data from persons across the lifespan within the Australian population, living in private residences by face-to-face interviews with trained staff of the ABS [15]. Detailed information on the Australian NHS methodology and methods have been previously published [14, 16, 17].

The overall sample included *n* = 20,460 persons [18]. For this study, only data from female participants aged 18 to 49 years were extracted from the ABS Confidentialised Unit Record File (CURF). For analysis these data were divided into three groups; pregnant women, breastfeeding women and non-pregnant/non-breastfeeding women (NP/NBF). No women in the sample reported they were currently pregnant while still breastfeeding. These sample data were then weighted to infer population estimates based on benchmarks established by the ABS using Australian Census data [14, 17]. This process is recommended by the ABS to ensure that survey results adhere to independent estimates of population distributions and can therefore be used to infer population estimates [14, 17].

Self-reported pain severity was defined as any bodily pain experienced during the previous 4 weeks on a six- point scale from ‘no pain’ to ‘very severe’ pain. Participants who did not answer this question were coded as having an ‘unknown’ or ‘not applicable’ response. These categories were classified into four groups for this study; no pain, very-mild-to-mild, moderate-to-very severe pain and not applicable or unknown. Self-rated health status was defined on a five-point scale from excellent to poor. Psychological distress was measured using the Kessler Psychological Distress Scale (K10), an established screening tool used to assess non-specific symptoms of psychological distress experienced over the most recent 30 days [19].

There are no universally accepted cut points for grouping K10 results [19]. ABS K10 scores are typically grouped into four levels: low distress (10–15); moderate distress (16–21); high distress (22–29); and very high distress (30–50) [19]. A strong association exists between a high K10 score and a current diagnosis of an anxiety or affective disorder using the Composite International Diagnostic Interview (CIDI) [20]. With sensitivity and specificity for the K10 assigned to scores along a continuous scale, a K10 score ≥ 16 has a sensitivity of 86% and specificity of 78% in identifying persons who meet CIDI criteria, a K10 ≥ 22 has a sensitivity of 55% and specificity of 95%, with 22 and 99% respectively for a K10 ≥ 30 [20]. For this study high and very high distress (K10 ≥ 22) were combined.

Analyses were weighted using the Jackknife delete-1 method to infer Australian population estimates, as recommended by the ABS for use with these data [17]. Fisher’s exact test for independence was used to test for associations with pain severity due to low cell frequencies in the pregnant and the breastfeeding women groups, with a chi-square test used for the non-pregnant/non-breastfeeding group. Frequencies were derived by applying the ABS population estimate proportions to the original sample size. A *p*-value of ≤0.05 was regarded as statistically significant.

## Results

This study included women aged 18 to 49 years who were pregnant (sample *n* = 165, weighted *N* = 191,856), breastfeeding (sample *n* = 210, weighted *N* = 234,601) or neither pregnant nor breastfeeding (sample *n* = 4005, weighted *N* = 4,607,140). Results for pain severity presented below may not equal 100% due to the exclusion of 0.2% of women in the NP/NBF group categorised as ‘not asked/not known’ and due to rounding.

### Prevalence of psychological distress in women of reproductive age by pain severity

Moderate-to-very severe pain was reported by 17.6% (95% CI, 9.5–25.9) of pregnant, 25.9% (95% CI, 18.9–32.8) of breastfeeding and 23.9% (95% CI, 22.2–25.6) of NP/NBF women. The majority of pregnant (52.6, 95% CI, 42.3–62.9), breastfeeding (40.5, 95% CI, 32.9–48.1) and NP/NBF women (43.7, 95% CI, 41.6–45.9) reported very-mild-to-mild pain. The percentage of women reporting no recent pain was similar across the three groups with a respective 29.7% (95% CI, 21.6%-37.9), 33.6% (95% CI, 25.3–41.9) and 32.1% (95% CI, 30.1–34.2) of pregnant, breastfeeding and NP/NBF women stating they had experienced no pain over the most recent four-week period.

The weighted point estimates for high-to-very high levels of psychological distress increased with pain severity, within each group of women (Table 1). Psychological distress was associated with pain severity in NP/NBF women (*p* < 0.001), but not in pregnant or breastfeeding women. High-to-very high distress levels were identified in 26.4% (95% CI, 23.2–29.6) of NP/NBF, 8.1% (95% CI, 0–17.2) of breastfeeding and 7.3% (95% CI, 0–18.0) of pregnant women with moderate-to-very severe pain.

**Table 1:** Psychological distress by pain severity in pregnant, breastfeeding and non-pregnant/non-breastfeeding women

	Pain Severity						
Psychological distress level	No recent pain		Very Mild to Mild		Moderate to Very Severe		*P* value
Pregnant (*n* = 165, *N* = 191,856)	(*n* = 52, *N* = 57,064)		(*n* = 86, *N* = 100,943)		(*n* = 27, *N* = 33,848)		
Low	82.9	(69.1–96.7)	78.8	(68.0–89.7)	71.4	(48.9–93.8)	
Moderate	15.1	(2.1–28.1)	14.5	(5.9–23.1)	21.3	(3.9–38.7)	0.550^a^
High to very high	2.0	(0.0–5.0)	6.7	(0.0–13.6)	7.3	(0.0–18.0)	
BF (*n* = 210, *N* = 234,601)	(*n* = 72, *N* = 78,889)		(*n* = 87, *N* = 95,019)		(*n* = 51, *N* = 60,694)		
Low	75.4	(61.8–89.0)	83.5	(74.6–92.4)	65.3	(48.3–82.4)	
Moderate	21.5	(8.8–34.1)	13.0	(5.7–20.3)	26.5	(9.5–43.6)	0.102^a^
High to very high	3.1	(0.0–8.8)	3.4	(0.0–7.4)	8.1	(0.0–17.2)	
Non-pregnant/non-BF (*n* = 4005*, *N* = 4,607,140)	(*n* = 1257, N = 1,481,078)		(*n* = 1725, N = 2,013,751)		(*n* = 1016, N = 1,100,795)		
Low	77.3	(74.2–80.4)	63.4	(60.1–66.6)	46.9	(42.5–51.4)	
Moderate	16.5	(14.1–18.9)	23.8	(21.1–26.4)	26.7	(23.1–30.2)	< 0.001^b^
High to very high	6.3	(4.6–7.9)	12.8	(10.9–14.8)	26.4	(23.2–29.6)	

Prevalence of psychological distress by gradation of pain severity. Prevalence as %, 95 confidence interval for estimate shown in parentheses. Psychological distress ranked by Kessler10 score (low: 10–15; moderate 16–21; high to very high: > 21). n: number of participants; N: inferred population estimate. *Seven women from the non-pregnant/non-BF group were categorised as not asked/not known and were not included. ^a^Fisher’s exact test ^b^Chi-square test. BF: Breastfeeding

### Self-rated health status in pregnant and breastfeeding women with pain

**Fig. 1:**
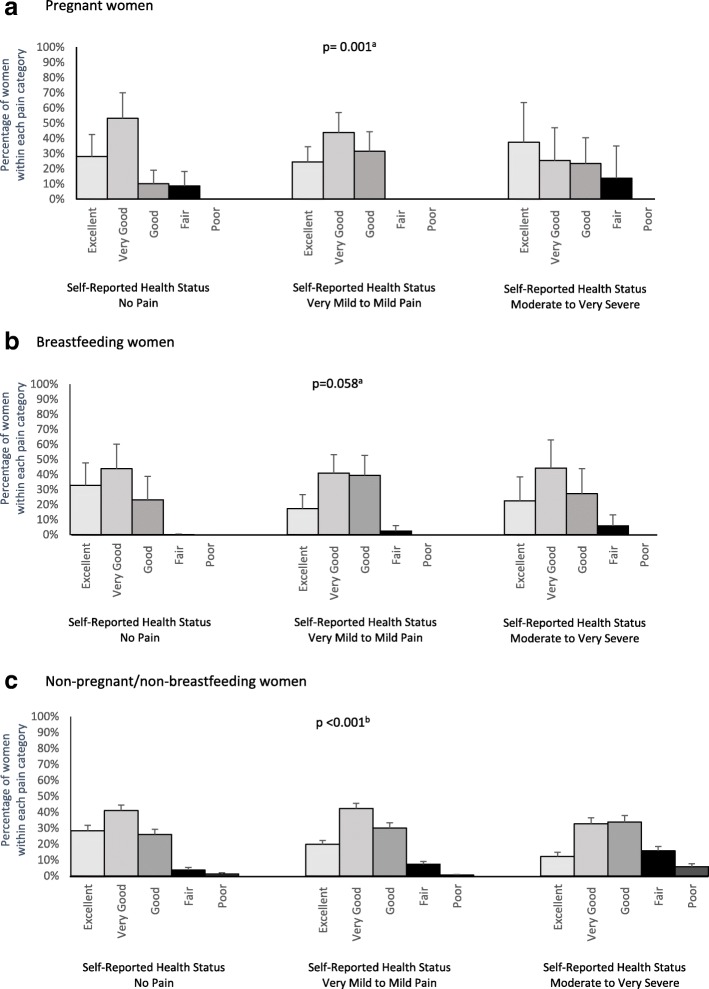
Distribution of women within each pain category by self-rated health status in **a**) pregnant women, **b**) breastfeeding women and **c**) non-pregnant/non-breastfeeding women, aged 18 to 49 years. ^a^Fisher’s exact test, ^b^Chi-square test. Error bars represent 95% confidence intervals

Figure 1 shows the distribution of self-rated health status for pregnant, breastfeeding and NP/NBF women by pain severity. Self-rated health status was associated with pain severity in pregnant (*p* = 0.001) and NP/NBF (*p* < 0.001) women, but not breastfeeding women (*p* = 0.058). In women with moderate-to-very severe pain, 15.8% (95% CI, 13.1–18.5) of NP/NBF, 6.0% (95% CI, 0–13.1) of breastfeeding and 13.8% (95% CI, 0–35) of pregnant women assessed their own health as ‘Fair’. The highest overall prevalence of both fair and poor health was reported by NP/NBF women with moderate-to-severe pain. Overall, many women rated their health as either ‘Excellent’ or ‘Very Good’, despite their experience of pain (Fig. 1 a, b and c). The main exception to this was seen in the NP/NBF women with moderate-to-very severe pain, where a small majority (33.7%) of women rated their health as ‘Good’ (Fig. 1c).

## Discussion

To our knowledge, this is the first study to assess psychological distress and self-rated health by pain severity in pregnant, breastfeeding and NP/NBF women. An association was found between psychological distress and pain severity in NP/NBF women and between self-rated health status and pain severity in both the pregnant and NP/NBF groups. Overall, high-to-very high psychological distress was most prevalent in women reporting moderate-to-very severe pain during the most recent four-week period.

Over one quarter (26.4%) of NP/NBF women with moderate-to-very severe pain were found to have high-to-very high psychological distress, more than three times higher than the point estimates reported for the pregnant and breastfeeding groups. It is unclear why the population point estimates for pain severity and psychological distress in the pregnant and breastfeeding groups were lower than for the NP/NBF group. It is possible that this may be reflective of differences in mean age or chronic health conditions between the groups of women. A previously published study using 2011–12 NHS data which investigated chronic pain prevalence and analgesia use in women aged 15–49 years reported a mean age of 30.2 years in the pregnant women and 32.3 years in non-pregnant women [5]. The same study found that there was a substantial difference in point estimates between these two groups of women regarding history of comorbidity related to mental wellbeing [5]. It may also be attributable to differences in perception of pain between the groups of women [21], with the expectation that pain related to pregnancy or breastfeeding will most likely resolve and is therefore accepted as normal, or to be expected. As with many surveys, other possibilities include recall or response bias. Response bias may be present where responses are selected because of perceived socially desirability, rather than accuracy [22], as may be the case with questions relating to psychological wellbeing [23].

In this study, NP/NBF women with moderate-to-very severe pain reported the highest prevalence of both fair and poor health. Low self-rated health is known to adversely impact future pregnancy intent in women of reproductive age, and is an established predictor of morbidity and mortality [2, 24]. However, previous assessments of self-rated health in pregnancy and in postpartum women have yielded inconsistent results. Studies which have investigated self-rated health status in pregnant and postpartum women have, for the most part, focused on health status in relation to socio-demographic factors or specific pregnancy-related conditions [25, 26]. Pain and painful conditions during pregnancy are often not reported in these studies, even though several recognize the common prevalence of pain in pregnant women [5, 10, 26] and the negative impact which it can have on quality of life during pregnancy [10]. From the available research, poorer self-rated health status during pregnancy has been associated with pain due to oral health conditions and low back pain [25, 27] as well as headache, low back pain, perineal pain and caesarean section within the first 2 months postpartum [26–28]. Whether the associations with caesarean section were directly related to pain resulting from the procedure, or a result of other factors is not known.

The strengths of this study include the use of nationally representative data collected in a methodologically rigorous manner by staff of the Australian Bureau of Statistics. This study also presents the most recent NHS data on pregnancy and breastfeeding, as these variables were not included in the survey conducted in 2014–15.

However, this study is not without limitations. As the Australian NHS relies mainly upon self-reported data, women may have been pregnant but unaware or may have declined to disclose their pregnancy when asked. Information on gestation or trimester of pregnancy was not available. We were also unable to assess maternal breastfeeding duration, nor directly define women who had recently given birth but who were not breastfeeding (e.g. formula-fed infants). Rather, the data for women who had recently given birth but were not breastfeeding would have been analysed in the NP/NBF group. Additionally, many factors impact upon psychological distress levels, and we were unable to ascertain the risk of increased prevalence of distress solely attributable to pain. Unfortunately, as the National Health Survey is undertaken to assess many aspects of health and illness in the Australian population, data collected specifically on factors related to pain and self-reported health status were limited. We were also limited in the statistical analyses which could be conducted due to restrictions of the ABS, the size of the pregnant and breastfeeding sample groups and limited data collected on potential confounding variables within the survey.

## Conclusion

This study presents findings on the prevalence of psychological distress and distribution of self-rated health status in Australian women of reproductive age, by pain severity. Overall, high-to-very high psychological distress was not uncommon in women of reproductive age with pain and was particularly common in NP/NBF women who reported moderate-to-very severe pain. It is known that high levels of psychological distress may be indicative of underlying anxiety and depression. It is also known that reducing psychological distress in pregnant and postpartum women is particularly important due to the potential impact upon maternal-infant attachment and future childhood development. Therefore, given that a strong association between psychological distress and pain in NP/NBF women was seen in this study, and that moderate-to-very severe pain was not uncommon in pregnant and breastfeeding women, assessment of psychological distress levels in any women of reproductive age who report experiencing moderate-to-very severe pain during the most recent four-week period may be beneficial.

## Abbreviations

ABS: Australian Bureau of Statistics
CIDI: Composite International Diagnostic Interview
CURF: Confidentialised unit record file
K10: Kessler 10 psychological distress scale
NHS: National Health Survey
NP/NBF: Non-pregnant/non-breastfeeding

## References

[1] Greenspan JD, Craft RM, LeResche L, Arendt-Nielsen L, Berkley KJ, Fillingim RB (2007). Studying sex and gender differences in pain and analgesia: a consensus report. Pain..

[2] Mynarska M, Wróblewska W (2017). The health of women of reproductive age and their childbearing intentions. Zdrowie Publiczne i Zarządzanie.

[3] Nahin RL (2015). Estimates of pain prevalence and severity in adults: United States, 2012. J Pain.

[4] Hassan S, Muere A, Einstein G (2014). Ovarian hormones and chronic pain: a comprehensive review. PAIN®..

[5] Miller April M., Sanderson Kristy, Bruno Raimondo B., Breslin Monique, Neil Amanda L. (2019). Chronic pain, pain severity and analgesia use in Australian women of reproductive age. Women and Birth.

[6] Korff MV, Simon G (1996). The relationship between pain and depression. Br J Psychiatry.

[7] Schug SA, Palmer GM, Scott DA, Halliwell R, Trinca J (2015). Acute pain management: scientific evidence. 4th ed.

[8] Kingston D, Tough S, Whitfield H (2012). Prenatal and postpartum maternal psychological distress and infant development: a systematic review. Child Psychiatry Hum Dev.

[9] Coluzzi F, Valensise H, Sacco M, Allegri M (2014). Chronic pain management in pregnancy and lactation. Minerva Anestesiol.

[10] Katonis P, Kampouroglou A, Aggelopoulos A, Kakavelakis K, Lykoudis S, Makrigiannakis A (2011). Pregnancy-related low back pain. Hippokratia..

[11] Eisenach JC, Pan PH, Smiley R, Lavand’homme P, Landau R, Houle TT (2008). Severity of acute pain after childbirth, but not type of delivery, predicts persistent pain and postpartum depression. Pain..

[12] Ding T, Wang DX, Qu Y, Chen Q, Zhu SN (2014). Epidural labor analgesia is associated with a decreased risk of postpartum depression: a prospective cohort study. Anesth Analg.

[13] Malhotra S, Khanna S (2016). Safety of analgesics in pregnancy. Int J Obstet Gynaecol Res.

[14] Australian Bureau of Statistics. Australian health survey: Users' guide, 2011–13 survey design and operation: Australian bureau of statistics. 2013. https://www.abs.gov.au/ausstats/abs@.nsf/Lookup/4363.0.55.001Chapter2002011-13. Accessed 30 Jul 2015.

[15] Australian Bureau of Statistics. Australian health survey: Users' guide, 2011–13 structure of the Australian health survey: Australian bureau of statistics. 2013. http://www.abs.gov.au/ausstats/abs@.nsf/Latestproducts/74D87E30B3539C53CA257BBB0014BB36?opendocument. Accessed 10 Oct 2015.

[16] Miller A, Sanderson K, Bruno R, Breslin M, Neil AL (2017). The prevalence of pain and analgesia use in the Australian population: findings from the 2011 to 2012 Australian National Health Survey. Pharmacoepidemiol Drug Saf.

[17] Australian Bureau of Statistics (2013). Australian health survey: Users' guide 2011–13 weighting, benchmarks and estimation procedures: Australian bureau of statistics.

[18] Australian Bureau of Statistics (2013). Australian health survey: Users' guide, 2011–13 response rates: Australian bureau of statistics.

[19] Australian Bureau of Statistics. Information Paper: Use of the Kessler Psychological Distress Scale in ABS Health Surveys, Australia, 2007-08. 2012. http://www.abs.gov.au/ausstats/abs@.nsf/Lookup/4817.0.55.001Chapter92007-08. Accessed 2017.

[20] Andrews G, Slade T (2001). Interpreting scores on the Kessler psychological distress scale (K10). Aust N Z J Public Health.

[21] Greenwood CJ, Stainton MC (2001). Back pain/discomfort in pregnancy: invisible and forgotten. J Perinat Educ.

[22] King MF, Bruner GC (2000). Social desirability bias: a neglected aspect of validity testing. Psychol Market.

[23] Wooden M. HILDA project discussion paper series: use of the kessler psychological distress scale in the HILDA survey. Melbourne: University of Melbourne; 2009.

[24] Idler EL, Benyamini Y (1997). Self-rated health and mortality: a review of twenty-seven community studies. J Health Soc Behav.

[25] Christian LM, Iams J, Porter K, Leblebicioglu B (2013). Self-rated health among pregnant women: associations with objective health indicators, psychological functioning, and serum inflammatory markers. Ann Behav Med.

[26] Semasaka JPS, Krantz G, Nzayirambaho M, Munyanshongore C, Edvardsson K, Mogren I (2016). Self-reported pregnancy-related health problems and self-rated health status in Rwandan women postpartum: a population-based cross-sectional study. BMC Pregnancy Childbirth.

[27] Haas JS, Jackson RA, Fuentes-Afflick E, Stewart AL, Dean ML, Brawarsky P (2005). Changes in the health status of women during and after pregnancy. J Gen Intern Med.

[28] Schytt E, Lindmark G, Waldenström U (2005). Physical symptoms after childbirth: prevalence and associations with self-rated health. BJOG..

